# The Treatment of Insomnia Secondary to Generalized Anxiety Disorder: Evaluating Escitalopram Use With Concomitant High-Resolution, Relational, Resonance-Based, Electroencephalic Mirroring (HIRREM)

**DOI:** 10.7759/cureus.45647

**Published:** 2023-09-20

**Authors:** Kinan Sawar, Gautham Pavar, Nicole Xu, Amar Sawar

**Affiliations:** 1 Plastic and Reconstructive Surgery, Wayne State University School of Medicine, Detroit, USA; 2 Anesthesia, Wayne State University School of Medicine, Detroit, USA; 3 Emergency Medicine, Wayne State University School of Medicine, Detroit, USA; 4 Neurology, Southern Illinois University School of Medicine, Springfield, USA

**Keywords:** electroencephalogram (eeg), hirrem, insomnia, health autonomy, escitalopram, generalized anxiety disorder (gad), insomnia severity index

## Abstract

Patient autonomy is important. However, what if a patient seeks out poorly studied treatment options? In this case report, we describe a patient with insomnia secondary to generalized anxiety disorder (GAD) who was prescribed escitalopram but additionally used High-Resolution, Relational, Resonance-Based, Electroencephalic Mirroring (HIRREM) therapy. HIRREM is an electroencephalogram (EEG)-based therapy that has been evaluated for use in the treatment of various conditions including insomnia. However, there has only been one randomized clinical trial supporting the use of HIRREM for insomnia, and the Food and Drug Administration (FDA) has not approved HIRREM for insomnia. A few months after the patient initiated HIRREM therapy and escitalopram cessation, the patient’s insomnia did not recur. We propose a case for how we approached educating a patient who was seeking out an alternative poorly tested therapy by helping him perform a cost-benefit analysis composed of treatment efficacy, cost, and side effects.

## Introduction

Patients often seek out inadequately studied medications and therapies to address their medical issues. For example, melatonin is commonly used for insomnia but is not formally prescribed in clinical practice. Another example is the use of chiropractic services by patients with chronic low back pain. In our patient’s case, he had been experiencing insomnia hampering his job as a truck loader for three weeks before deciding to seek medical attention. He was started on escitalopram, an established treatment for insomnia. However, he also independently sought out High-Resolution, Relational, Resonance-Based, Electroencephalic Mirroring (HIRREM) therapy, a poorly studied treatment for insomnia.

HIRREM involves placing a patient in a dark room, wiring them to an electroencephalogram (EEG) machine, and providing them with a pair of headphones to wear. The EEG captures the amplitudes of various brain frequencies, which are then analyzed by proprietary software to generate sounds that mirror these brain frequencies. These sounds are then delivered to the patient via the headphones. The proponents of HIRREM claim that hearing these mirrored auditory tones allows the brain to auto-calibrate itself toward physiologically normal brain frequencies. The processing of synchronized EEG waveforms and the production of auditory tones occur within a few milliseconds, allowing this process to be nearly real time. This therapeutic modality involves patients undergoing 90-minute audio sessions for a total of 10 sessions. HIRREM has preliminary evidence for the treatment of insomnia, post-traumatic stress disorder, and traumatic brain injury [1-3]. Ongoing clinical trials are currently assessing the use of this technology for the treatment of primary hypertension, hot flashes, and anxiety, among other conditions [1].

However, seeking out this treatment based solely on this preliminary evidence has a few issues. Firstly, there is limited evidence supporting the use of HIRREM for the treatment of insomnia per our literature review. Secondly, this treatment may not be covered by insurance, placing a considerable financial burden on patients who opt for this therapy. Lastly, it is crucial to note that the only published clinical trial evaluating HIRREM’s use for treating insomnia has significant conflicts of interest. The researchers conducting the study also have an ownership stake in a business called Brain State Technologies, which utilizes the same technology [1].

The patient’s insomnia resolved while he was following escitalopram and HIRREM treatment. However, his use of HIRREM resulted in significantly higher medical expenses with uncertain benefits. The healthcare provider team educated the patient about the data supporting escitalopram’s use in generalized anxiety disorder (GAD)-induced insomnia, as well as the lack of evidence supporting HIRREM for this indication. Despite this education, the patient chose to use HIRREM in addition to escitalopram, believing that it would be effective for him. When discussing different treatment options with patients, maintaining objectivity is crucial to respecting patient autonomy. The healthcare team proactively educated the patient about these different treatments while still ultimately respecting the patient’s decision to pursue the alternative treatment modality. The primary lesson from this case is that clinicians should conduct a cost-benefit analysis when discussing treatment modalities with patients to ensure that patients can make informed decisions when the treatments may have limited efficacy, high costs, or numerous unwanted side effects.

## Case presentation

A man in his 40s presented to our primary care clinic for insomnia due to patient restlessness that occurred almost every night for three weeks, awakening him from sleep most nights. Our first step was to evaluate the cause of this patient’s insomnia.

The differential diagnosis for insomnia is broad but was easily narrowed down in this patient’s case because of his lack of past medical history. Because this patient only experienced symptoms for three weeks, which is less than the three months required for a chronic insomnia diagnosis, his diagnosis was that of acute insomnia. The social determinants of health for our patient included working a nighttime job and living at home with parents. His body mass index (BMI) was 18.10 kg/m^2^, and he had a normal vitamin B12 level at 496 pg/mL (normal range: 200-1,000 pg/mL), a normal thyroid-stimulating hormone (TSH) level at 2.16 mU/L (normal range: 0.4-4.0 mU/L), and a normal brain magnetic resonance imaging (MRI) study. Our differential diagnosis included hyperthyroidism, multiple sclerosis, anxiety-induced insomnia, depression-related insomnia, shift work sleep disorder, and delayed sleep-wake phase disorder.

We were able to quickly rule out hyperthyroidism, multiple sclerosis, and other primary brain pathologies based on the patient’s normal TSH and MRI. Shift work sleep disorder was ultimately ruled out as the patient had been participating in night shifts for many months before the symptom onset. Delayed sleep-wake phase disorder was ruled out during history-taking as the patient revealed that sleep timing was not necessarily an issue. The patient did not meet the Diagnostic and Statistical Manual of Mental Disorders, Fifth Edition (DSM-5) diagnostic criteria for major depressive disorder, so depression-related insomnia was ruled out. Notably, the patient met DSM-5 criteria for GAD as he experienced anxiety, restlessness, fatigue, difficulty concentrating, irritability, and sleep disturbances. Thus, insomnia secondary to generalized anxiety disorder was diagnosed.

At the patient’s initial visit, clonazepam 0.5 mg was prescribed twice daily for symptomatic management. Four weeks later, the patient followed up with our team and reported a significant improvement in his sleep, including a return to his baseline sleep length and continuity. During this follow-up visit, the patient’s treatment was changed from clonazepam to escitalopram 5 mg daily for the long-term management of his GAD.

Six weeks later, the patient returned for a follow-up appointment and reported continued improvement in his sleep, as well as an overall sense of contentment, both physically and emotionally. His mother also stated that he had been interacting with family members more frequently and no longer complained of sleep issues. At this time, the patient was still taking escitalopram 5 mg by mouth once daily. However, the patient stated that he also started attending sessions at an institute that provides HIRREM therapy around one month after starting taking escitalopram. At the time of this follow-up visit, the patient had undergone five HIRREM sessions and had already paid for an additional 11 sessions to be scheduled shortly.

Three months later, the patient was seen again for a follow-up appointment. At this point, he had completed his HIRREM sessions to the tune of $1,100, per the patient. During this visit, the patient denied ever experiencing any common adverse effects associated with escitalopram such as diarrhea, drowsiness, gastrointestinal disturbance, rash, insomnia, impotence, or headaches. Given the medication’s efficacy, we decided to continue the same dosage of escitalopram, monitor for any adverse effects, and schedule a follow-up appointment in three months to monitor the patient’s progress.

**Figure 1:**
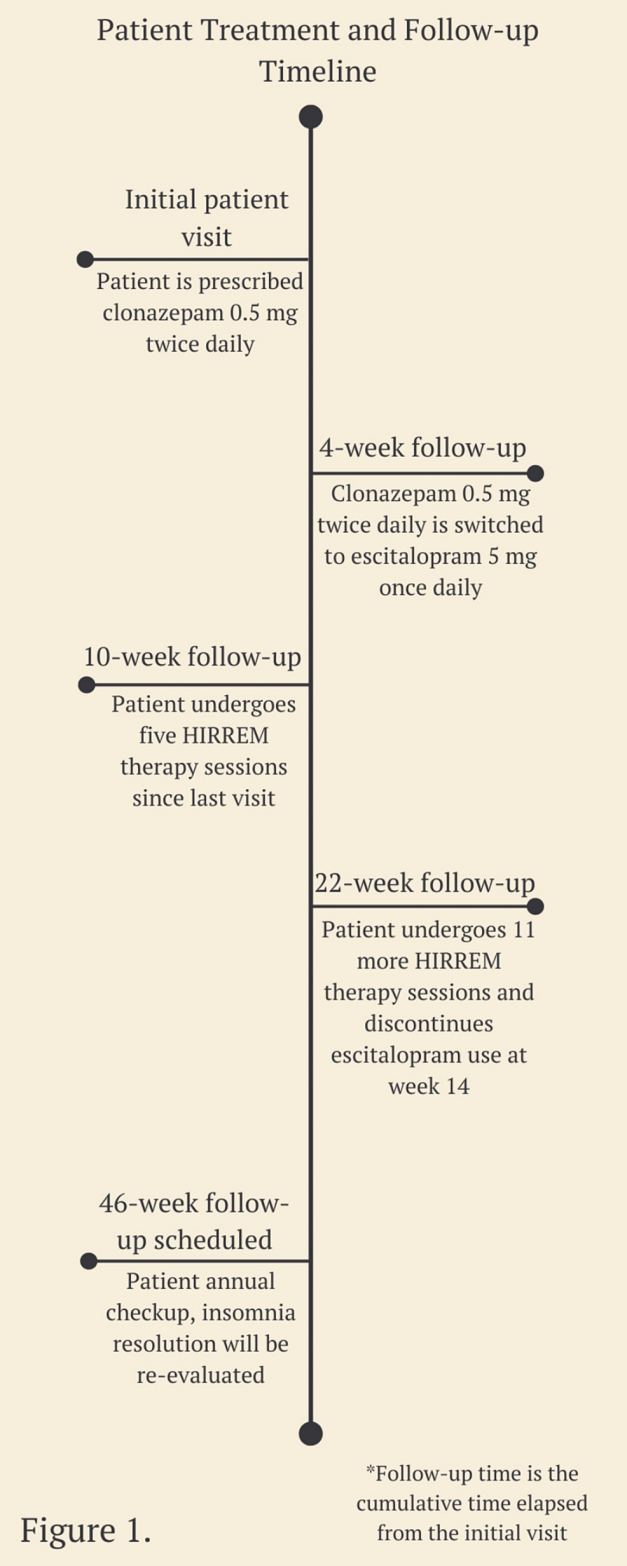
Patient treatment and follow-up timeline HIRREM: High-Resolution, Relational, Resonance-Based, Electroencephalic Mirroring

At this next visit, the patient reported that he had discontinued taking escitalopram two months ago and had not observed any decline in his sleep quality. He reported sleeping eight hours a night consistently. Considering that the patient had gone through two months without escitalopram, our team deemed it appropriate to avoid restarting the medication and rather schedule a six-month follow-up to reassess the patient’s progress. The entire patient treatment and follow-up timeline can be referred to in Figure 1.

## Discussion

Diagnosing chronic insomnia requires the symptom of difficulty sleeping for three nights a week for a minimum of three months and can be confirmed with neuroimaging, which was not available to the care team [4]. Our patient presented with acute insomnia since he had only been experiencing the symptoms for the past three weeks.

It remains unclear whether the patient’s clinical improvement was due to escitalopram, HIRREM, or a combination of both. This is because the patient had started HIRREM therapy around the same time that selective serotonin reuptake inhibitors (SSRIs) typically begin to reach greater efficacy (one month after initiation) [5]. Although SSRIs can begin to show signs of clinical efficacy after just one to two weeks [5], the patient was uncertain whether he noticed any significant improvement in symptomatology until the last two weeks, which happened to coincide with his attendance at HIRREM therapy sessions. Our care team decided to evaluate the literature on the use of HIRREM for acute insomnia to provide us with information on approaching the care of this patient.

A PubMed search for case studies utilizing HIRREM’s use in the treatment of insomnia yielded no results. To our knowledge, this case study is the first of its kind to discuss the use of HIRREM for any medical indication.

There is sparse documented clinical use of HIRREM. However, there has been a single 107-patient clinical trial done evaluating the efficacy of HIRREM for patients experiencing insomnia [1]. Patients were divided into two groups: one group received the treatment, while the other received a sham placebo in which random acoustic sounds were played that did not correspond to the patient’s EEG activity. Participants were middle-aged males and females, predominantly Caucasian. Patients were excluded from the study if they were actively taking selective serotonin reuptake inhibitors (SSRIs), serotonin and norepinephrine reuptake inhibitors (SNRIs), tricyclic antidepressants (TCAs), opiates, antipsychotics, or benzodiazepines.

Half of the patients in this study were treated with HIRREM four times in the first two days, two more times in the first week, and four more times over the next two weeks for a total of 10 treatments over three weeks. All primary and secondary measures were collected at four time points: at baseline, after one week, averaged over weeks 8-10, and averaged over weeks 16-18. The study showed that using HIRREM to treat insomnia resulted in moderate improvement in patient-reported symptoms related to insomnia including improvement in self-reported blood pressure, heart rate, heart rate variability, depression, and anxiety in quality of life questionnaires. Insomnia improvement was directly demonstrated by reduced patient insomnia severity index (ISI) scores. No severe adverse effects were reported [1].

This study utilized the ISI to determine the clinical response to insomnia treatment. ISI scores have been shown to be a valid and sensitive measure to detect changes in perceived sleep difficulties [6]. The authors utilized a score greater than 15 as the sole criterion for a clinical diagnosis of insomnia. An ISI score of 10 or higher has been shown to have a sensitivity of 86.1% and specificity of 87.7% for the diagnosis of insomnia [7]. The ISI ranges from a score of 0 to 28. The ISI is an assessment of seven insomnia characteristics on a five-point Likert scale, where 0 is the absence of a particular problem and 4 indicates the maximum severity of that problem [7]. After adding up the points from the individual characteristics, clinicians can categorize the severity of insomnia: 0-7 implies the absence of insomnia, 8-14 subthreshold insomnia, 15-21 moderate insomnia, and 22-28 severe insomnia. Morin et al. also found statistically significant associations between a decrease in ISI scores and improvement in patient-reported clinical symptoms of insomnia: “slight improvement” in insomnia corresponded to a 4.65-point decrease in the ISI score; likewise, “moderate improvement” corresponded to an 8.36-point decrease, and “marked improvement” corresponded to a 9.89-point decrease [7].

The use of HIRREM for insomnia only showed a moderate improvement of insomnia symptoms with a reported improvement of 7.93 points on the ISI scale. This is similar to the average score decrease of 8.36 that Morin et al. use to characterize a moderate improvement in insomnia [1,7]. The data also showed that the sham control provided a slight improvement in insomnia symptomatology with a corresponding decrease of 5.90 points, providing evidence that a sham EEG-based therapy could even be used as an effective placebo for the treatment of insomnia. Lastly, HIRREM’s use for anxiety and depression has shown to be statistically insignificant, complicating the case for a patient who is suffering from anxiety-induced insomnia [1].

Additionally, the authors advocated for HIRREM’s use in insomnia treatment because it decreased a patient’s heart rate variability, a once commonly used indicator to monitor insomnia treatment [1,8]. However, the most up-to-date literature reports that heart rate variability is a poor marker for insomnia treatment progress [8]. This information weakens the argument for HIRREM’s use in the treatment of insomnia [1,8].

Although HIRREM showed clinically significant improvement of patients’ insomnia symptoms, there was no statistically significant impact on patient-reported symptoms of anxiety. Our patient was suffering from acute insomnia secondary to GAD; this along with the confounding effect of escitalopram on our patient’s clinical improvement makes it difficult to assess HIRREM’s efficacy. Nevertheless, there are many other proven medications and therapies for the treatment of acute anxiety-induced insomnia that would likely be superior to HIRREM. For example, benzodiazepines, benzodiazepine receptor agonists, and antidepressants have been shown to provide moderate improvement in acute insomnia symptoms based on a reduction in ISI score [9]. If a patient is considering HIRREM therapy for acute insomnia, these other options should at least be recommended to the patient given their acceptance by the Food and Drug Administration (FDA) and insurance companies and in the literature [9].

## Conclusions

In summary, here are some learning points to take away from this case presentation. There are better alternatives to HIRREM for the treatment of acute anxiety-induced insomnia, including benzodiazepines, benzodiazepine receptor agonists, and antidepressants. If patients pursue treatment modalities that the physician is unfamiliar with, it is crucial to review reputable published research that may be available regarding that treatment modality. If a dual or multimodal treatment is effective, it may be reasonable to maintain it. In special circumstances, non-FDA-approved drugs, placebo drugs, and sham treatments may be clinically beneficial for patients to continue, and some patients come to the clinic already actively utilizing these nontraditional options. However, the patient should be properly informed of the efficacy, cost, and possible adverse side effects of their choice. The healthcare provider should advise against the treatment option if either the side effect profile is unknown or there is a substantial risk of negative outcomes to patient health. The efficacy of individual treatment modalities can be difficult to evaluate whenever a patient is on dual therapy or multi-therapy that has not been studied, even if one or both of the individual treatments have been well studied, so clinical judgment is warranted in these cases to properly educate patients as to their possible options.
